# Combined application of latissimus dorsi myocutaneous flap and iliac bone flap in the treatment of chronic osteomyelitis of the lower extremity

**DOI:** 10.1186/s13018-018-0824-z

**Published:** 2018-05-18

**Authors:** Jihui Ju, Lei Li, Rong Zhou, Ruixing Hou

**Affiliations:** Department of Hand Surgery, Ruihua Affiliated Hospital of Soochow University, No. 5 Tayun Road, Suzhou, 215104 China

**Keywords:** Bone defects, Chronic osteomyelitis, Flap grafting, Microsurgical technique

## Abstract

**Background:**

To evaluate the clinical efficacy and safety of latissimus dorsi myocutaneous flap (stage I) combined with iliac bone flap (stage II) in the treatment of chronic osteomyelitis of the lower extremity.

**Methods:**

Clinical data of 18 patients undergoing latissimus dorsi myocutaneous flap in combination with iliac bone flap grafting were retrospectively analyzed. Among them, 2 patients developed chronic osteomyelitis of the lower segment of the femur, 4 were diagnosed with chronic osteomyelitis of the tibial plateau, and 12 with chronic osteomyelitis of the lower segment of the tibia.

**Results:**

All the latissimus dorsi myocutaneous flaps survived in 18 patients. After the corresponding surgery, primary wound healing was achieved in 11 patients, and delayed wound healing was obtained in 7 cases. All wounds were completely healed with postoperative 2 months. Following the iliac bone flap grafting, primary would healing was accomplished in all cases. All dorsal window chambers survived. The bone defects were properly restored within 4–12 postoperative months. Functional training was performed after removal of the internal and external fixators. Postoperative follow-up was endured from 6 months to 10 years. All patients were satisfied with the bone healing and flap texture without the incidence of osteomyelitis and sinus tract. No contraction was observed in the grafting area of 2 patients receiving latissimus dorsi myocutaneous flap grafting. Residual linear scars were noted in the dorsal and iliac donor sites.

**Conclusion:**

Combined usage of stage I latissimus dorsi myocutaneous flap and stage II iliac bone flap grafting is an efficacious and safe surgical technique in clinical practice.

## Background

Osteomyelitis is an infection of the bone and bone marrow. It may be subdivided into acute, sub-acute, and chronic stages. Chronic osteomyelitis is a frequent complication after compound fractures of the lower extremity. It may appear as such at the initial stage, and not all patients present with disease progression through the three stages. Sclerotic non-purulent form of osteomyelitis rarely occurs. Alternative types of disorders are chronic recurrent multifocal osteomyelitis, tuberculous osteomyelitis, synovitis, acne, pustulosis, hyperostosis, and osteitis syndrome. The key procedure throughout the treatment is the radical debridement of all scar tissue, muscle, and infected bone. It constantly causes large tissue defects, which especially in the distal third of the tibia can only be sutured with free tissue flap and re-vascularized by a microvascular anastomosis. At present, surgical interventions for chronic osteomyelitis primarily include persistent fenestration and lavage muscle flap filling, antibiotic bone cement filling and bone resection, etc. [1–4]. All these approaches have been proven to yield relatively high clinical efficacy in specific cases. Nevertheless, the enduring course of treatment is extremely long, even exceeding dozens of years, which significantly affects the quality of life and poses non-tolerable economic burden to the patients [5].

The concealed donor site and excellent texture of latissimus dorsi myocutaneous flap gain clinical advantages. In addition, the fixed anatomical position of blood vessels, thick vascular diameter and abundant blood supply, and high anti-infection capability allow for proper application in the treatment of multidrug resistant bacteria of the lower extremity in clinical practice [6–8]. Iliac bone flap also possesses multiple merits, such as constant nutrient vessels, thick vascular diameter, and high content of spongy bone for convenient resection and remodeling. The donor site is hideous and yields slight postoperative complications, which is an ideal donor site for bone defect grafting. Currently, iliac bone flap grafting is widely applied in the repairing of bone defects in clinical settings [9, 10]. In this investigation, 18 patients diagnosed with chronic osteomyelitis of the lower extremity undergoing stage I latissimus dorsi myocutaneous flap combined with stage II iliac bone flap grafting between June 2004 and December 2014 were recruited. The clinical efficacy and safety of the combined surgical interventions were thoroughly evaluated and reported as below.

## Methods

### Baseline data

From June 2004 to December 2014, 18 patients diagnosed with chronic osteomyelitis of the lower extremity were enrolled in this investigation, including 16 males and 2 females, aged 23–65 years, 47 years on average. The main causes of chronic osteomyelitis of the lower extremity included traffic accident wound in 11 cases (61.1%) and machine twist injury in the remaining 7 (38.9%). Written informed consents were obtained from all patients. The study procedures were approved by the ethics committee of Ruihua Affiliated Hospital of Soochow University.

### Chronic osteomyelitis site

Two patients were diagnosed with chronic osteomyelitis of the lower segment of the femur, 4 cases (22.2%) with chronic osteomyelitis of the tibial plateau and 12 patients (66.7%) with chronic osteomyelitis of the middle and lower segments of the tibia. The time interval between onset of injury and clinical treatment was ranged from 1 to 15 years, 3.2 years on average. The cycle of clinical treatment endured from 11 months to 3 years. During the stage I intervention, the bone lesions were thoroughly debrided and the necrotic cavities were filled with latissimus dorsi myocutaneous flap. After 6 to 10 months, the stage II treatment was delivered and the bone defects were repaired by using the iliac bone flap grafting. The bone defects were measured from 4 to 10 cm in length, 6 cm on average. The size of latissimus dorsi myocutaneous flap was approximately from 8 cm × 5 cm to 18 cm × 12 cm. In 10 patients, the iliac bone flaps were accompanied with skin window chamber of 6 cm × 4 cm to 10 cm × 6 cm in area. The length of the ilial bone was measured from 5 to 11 cm, 6 cm on average, as illustrated in Table 1.

**Table 1:** Summary of baseline data in 18 patients

No.	Fracture site	Injury date	Cause of injury	Bacterial culture results	Latissimus dorsi myocutaneous flap area	Iliac flap (months)	Bone defect length	Skin window chamber area
1	Left tibiofibula	2015/4/14	Traffic accident injury	*Enterobacter cloacae*	8 cm × 5 cm	13	4	6 cm × 4 cm
2	Right tibiofibula	2013/8/3	Traffic accident injury	*Enterobacter cloacae*	10 cm × 7 cm	24	5	6 cm × 5 cm
3	Left tibia	2014/12/4	Machine injury	*Staphylococcus aureus*	11 cm × 7 cm	11	5	7 cm × 4 cm
4	Right tibiofibula	2012/9/3	Traffic accident injury	*Enterococcus faecium*	10 cm × 6 cm	14	6	–
5	Right tibiofibula	2009/12/31	Traffic accidents injury	*Staphylococcus aureus*	16 cm × 6 cm	28	6	11 cm × 5 cm
6	Left tibia	2011/7/10	Machine injury	*Pseudomonas aeruginosa*	12 cm × 9 cm	11	5	7 cm × 5 cm
7	Right tibia	2013/4/11	Traffic accident injury	*Staphylococcus aureus*	14 cm × 5 cm	12	6	9 cm × 4 cm
8	Right tibiofibula	2015/4/10	Traffic accident injury	*Escherichia coli*	18 cm × 12 cm	11	8	–
9		2014/8/12	Traffic accident injury	*Enterococcus faecium*	10 cm × 7 cm	16	6	6 cm × 5 cm
10	Left tibiofibula	2012/7/11	Traffic accident injury	*Staphylococcus aureus*	14 cm × 8 cm	18	5	7 cm × 5 cm
11	Right tibia	2013/3/14	Machine injury	*Pseudomonas aeruginosa*	12 cm × 7 cm	13	7	7 cm × 5 cm
12	Right tibia	2014/6/4	Traffic accident injury	*Enterococcus faecium*	16 cm × 8 cm	19	6	8 cm × 4 cm
13	Right tibia	2014/1/9	Machine injury	*Enterobacter cloacae*	15 cm × 8 cm	18	5	9 cm × 4 cm
14	Left tibiofibula	2015/1/12	Traffic accident injury	*Staphylococcus aureus*	10 cm × 5 cm	20	6	7 cm × 5 cm
15	Right tibiofibula	2012/12/21	Machine injury	*Staphylococcus aureus*	9 cm × 7 cm	18	7	8 cm × 4 cm
16	Left tibia	2013/11/12	Machine injury	*Enterobacter cloacae*	13 cm × 5 cm	15	6	8 cm × 5 cm
17	Left tibiofibula	2014/8/6	Traffic accident injury	*Escherichia coli*	14 cm × 8 cm	12	4	6 cm × 5 cm
18	Right tibiofibula	2013/7/7	Traffic accident injury	*Staphylococcus aureus*	13 cm × 6 cm	11	6	9 cm × 5 cm

### Preoperative preparations

Prior to corresponding interventions, X-ray and CT scan were performed. In addition, bacterial culture was delivered. Regional medullary cavity sclerosis with indistinct margin was detected. The formation of necrotic bone was observed in regional bone defects. Alternative chronic osteomyelitis-related symptoms including inflammatory callus, increase in bone density, and recurrent sinus tract also occurred.

### Surgical procedures

#### Preoperative preparation

Surgical procedures were specifically designed according to preoperative parameters, such as the site, severity and range of chronic osteomyelitis, the severity of soft tissue defects, and the presence of anastomosis-supplying blood vessels. Prior to surgery, the arteries of the lower extremity were pinpointed by using color Doppler ultrasound to identify the anastomosis of the flap blood vessels. The secretions in the wound or sinus tract were collected and subject to bacterial culture and drug sensitivity test. Sensitive antibiotics were chosen to deliver effective anti-infection treatment.

#### Debridement and surgical removal of bone defects

A portion of 20–50 ml diluted methylene blue was slowly injected into the sinus tract or wound. The skin adjacent to the sinus tract was resected and the area with skin pigment change was also excised extending to the deep tissues. The affected skin tissues stained by methylene blue were thoroughly resected. Both necrotic and sclerosis bone defects were removed until evident errhysis was observed. The medullary cavity stained with methylene blue was thoroughly debrided. After repeated lavage, operative instruments were replaced, the operative tables were arranged, and bone defect sections were treated smooth and the surface of the flaps was specifically designed.

#### Resection and repairing of latissimus dorsi myocutaneous flaps

The skin incisions were created along with the anterior margin of the armpit latissimus dorsi. The thoracodorsal artery and nerve were identified and extended to the perforating muscular branches. The muscular flaps were created along with the plane of perforating muscular branches. Partial latissimus dorsi was resected according to the required quantity of the muscle for the wound surface to prevent the separation between the muscle and skin. After distal tissue excision, retrograde anatomical resection was performed to the armpit thoracodorsal artery. The status of the muscular and skin blood supply was observed. For patients with evident errhysis in the muscular and skin margin, the pedicle was cut at a high position and the donor site was directly sutured. For those unsuitable for direct suture, full-thickness skin grafting was performed on the wound surface. The iliac crest flap needs higher flap raising expertise than other flaps as previously described [11].

#### Resection and repairing of iliac bone flaps

The flap design and marking were performed according to the bone defect length. The pulsation site of femoral artery was regarded as the starting point and the anterior superior iliac spine was considered as the end point. A connecting line was drawn between two points. During the cutting of iliac bone flaps, the iliac blood vessel branches were properly protected and the separation between vessel pedicle and iliac bone was averted as possible. The iliac bone was treated smooth and sealed with bone wax. The donor site was subject to direct suturing. The vessels distant from the lesions were chosen for vascular anastomosis. Two patients presented with chronic osteomyelitis of the lower segment of the femur, 4 with chronic osteomyelitis of the tibial plateau and 12 with chronic osteomyelitis of the middle and lower segments of the tibia.

### Postoperative processing

Postoperatively, the latissimus dorsi myocutaneous flaps were subject to routine anti-infection, anti-coagulation, and anti-spasm interventions. Anti-coagulation protocols: irrigation with a 250-U/ml (6250 U heparin/250 ml normal saline) solution of heparin, intravenous drip, once a day. Aspirin and dipyridamole were administered per os, once 25 mg, three times daily. The duration of anti-coagulation therapy was 1 week. Sensitive antibiotics were administered for anti-infection therapy for 3 weeks. The wound dressing was changed once every 2 days. During dressing change, the hematocele beneath the flaps were squeezed and the suture line was removed timely. Patients were discharged from hospital after wound healing. Postoperative follow-up was performed on a regular basis. Following iliac bone flap grafting, routine anti-infection, anti-coagulation, and anti-spasm interventions were also delivered. At postoperative 12 days, all patients were recovered and successfully discharged from hospital. Postoperative and discharge data of the patients were collected during subsequent follow-up.

## Results

### Overall surgical efficacy

All latissimus dorsi myocutaneous flaps survived. Primary wound healing was achieved in 11 patients and delayed wound healing was obtained in the remaining 7 cases. All delayed wounds were healed within 2 months. Following iliac bone flap grafting, primary wound healing was obtained in 18 cases. For 10 cases, the flaps accompanied with skin window chamber all survived. The bone defects were fully healed at postoperative 4–12 months. Patients were required to perform functional training after the removal of internal and external fixators. Postoperative follow-up endured from 6 months to 10 years.

### Overall postoperative complications

Excellent bone healing was obtained. The flap texture was qualified. Neither recurrent osteomyelitis nor sinus tract occurred. No contraction was observed in the grafting area of 2 patients receiving latissimus dorsi myocutaneous flap grafting. Residual linear scars were noted in the dorsal and iliac donor sites. Paley’s criterion was adopted to assess the bone healing and function recovery of the lower limbs. Excellent bone healing was accomplished in 13 patients and good bone healing was obtained in the remaining 5 cases. In terms of the function recovery, 12 patients obtained excellent effect, 5 had good effect, and 1 obtained moderate effect.

### Case presentation

**Fig. 1:**
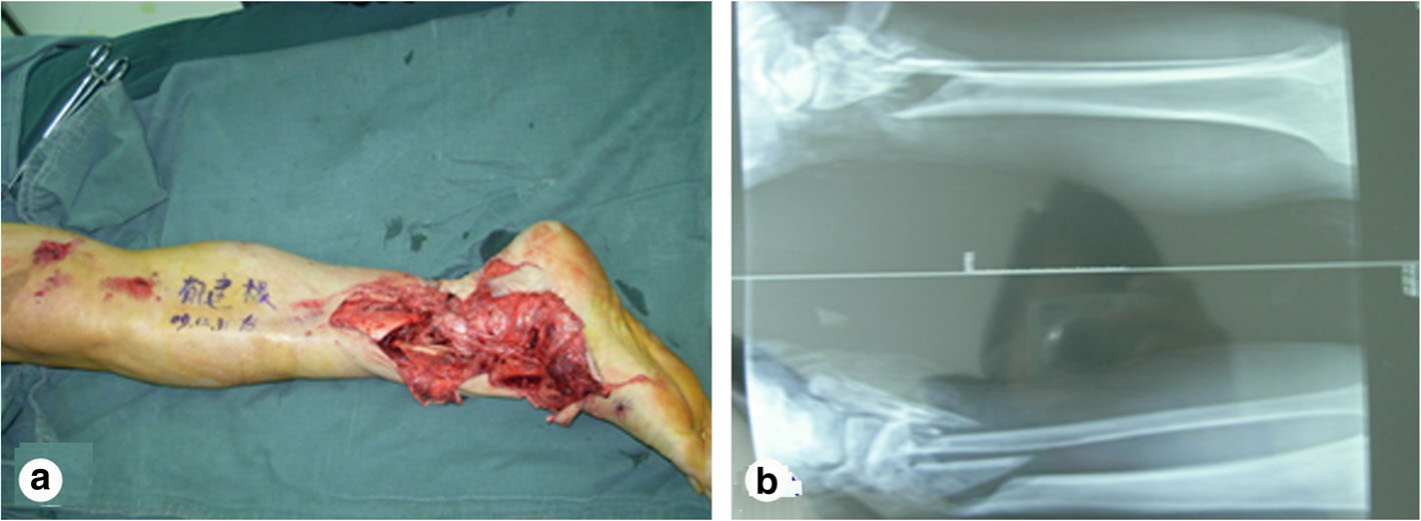
Open injury of the right lower extremity (**a**). Tibiofibular comminuted fracture of the right lower extremity (**b**)

**Fig. 2:**
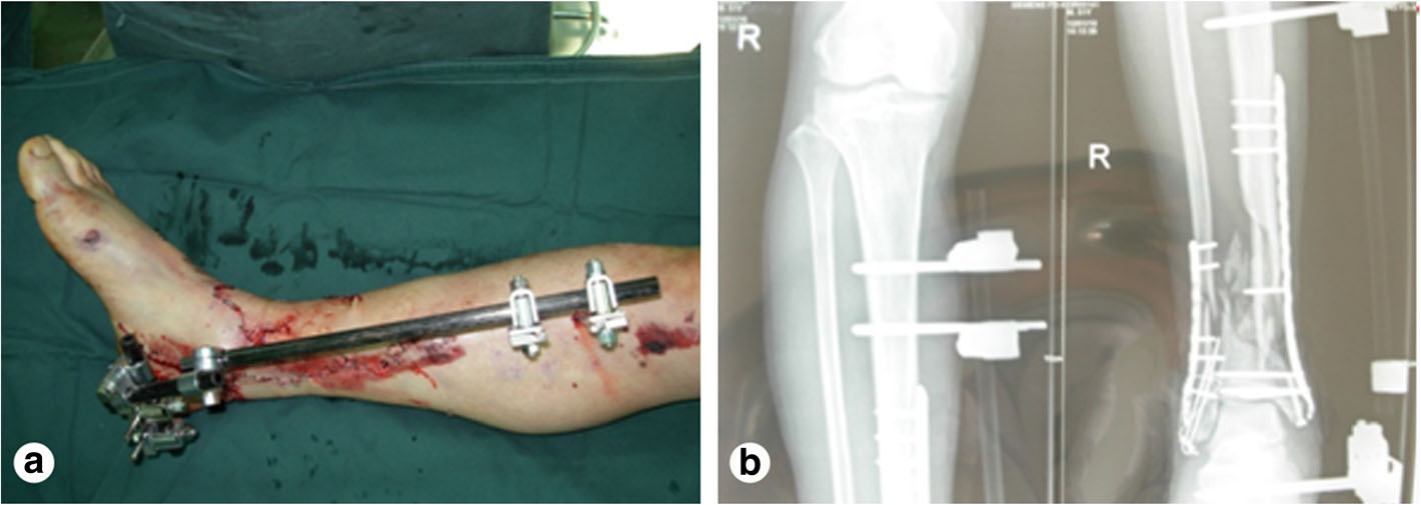
Emergent surgery (**a**) and emergent internal fixation (**b**)

**Fig. 3:**
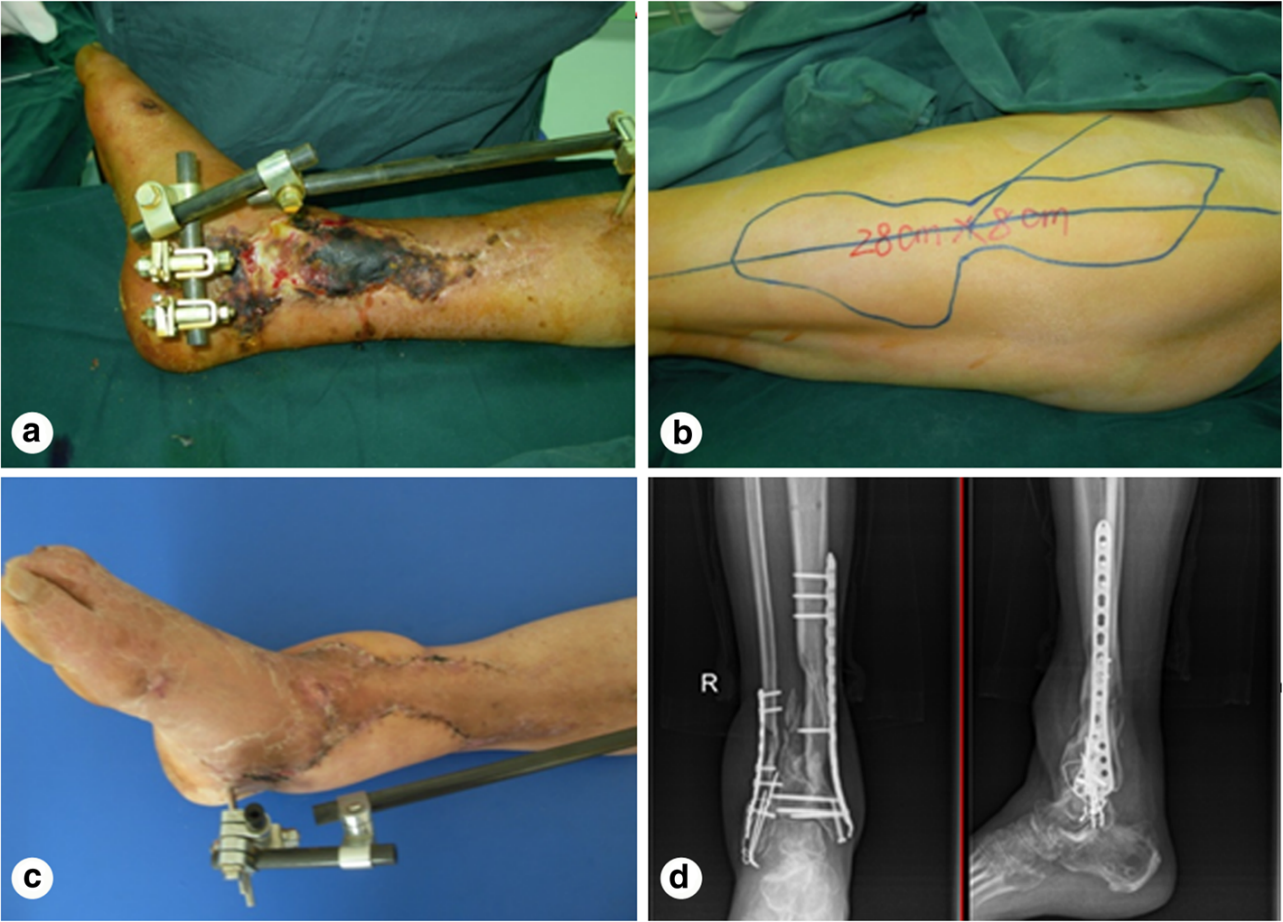
Partial skin necrosis in the ankle (**a**). Lateral flap design of the anterior femur (**b**). Wound surface was covered by lateral flap of the anterior femur (**c**). External fixation stent is removed (**d**)

**Fig. 4:**
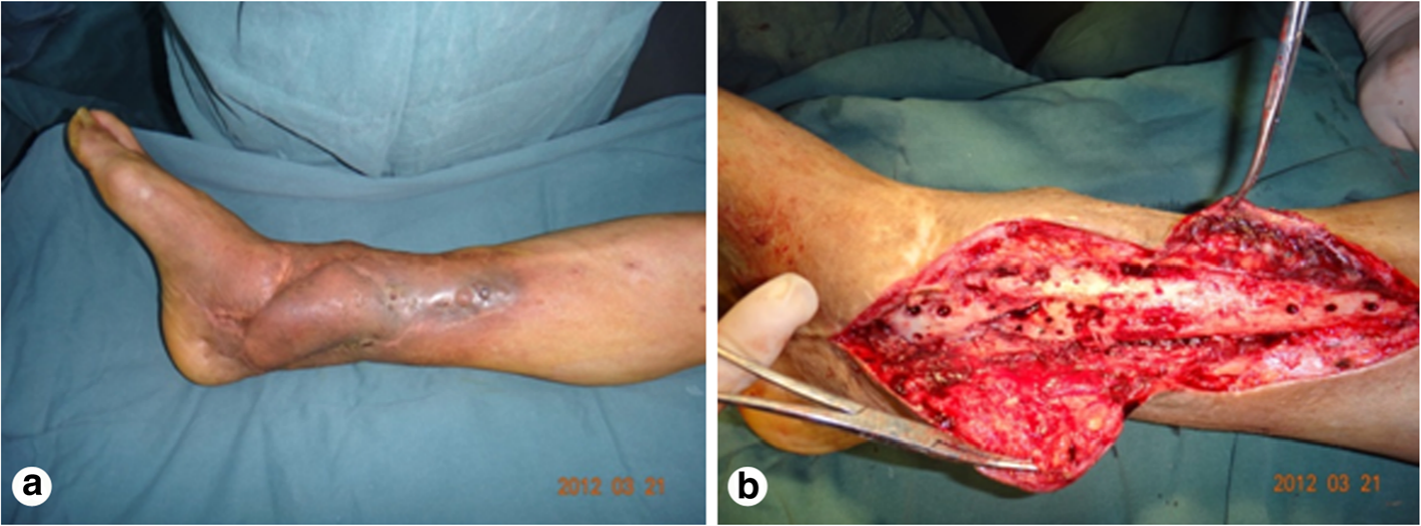
Incidence of the chronic osteomyelitis (**a**). Internal fixator is removed (**b**)

**Fig. 5:**
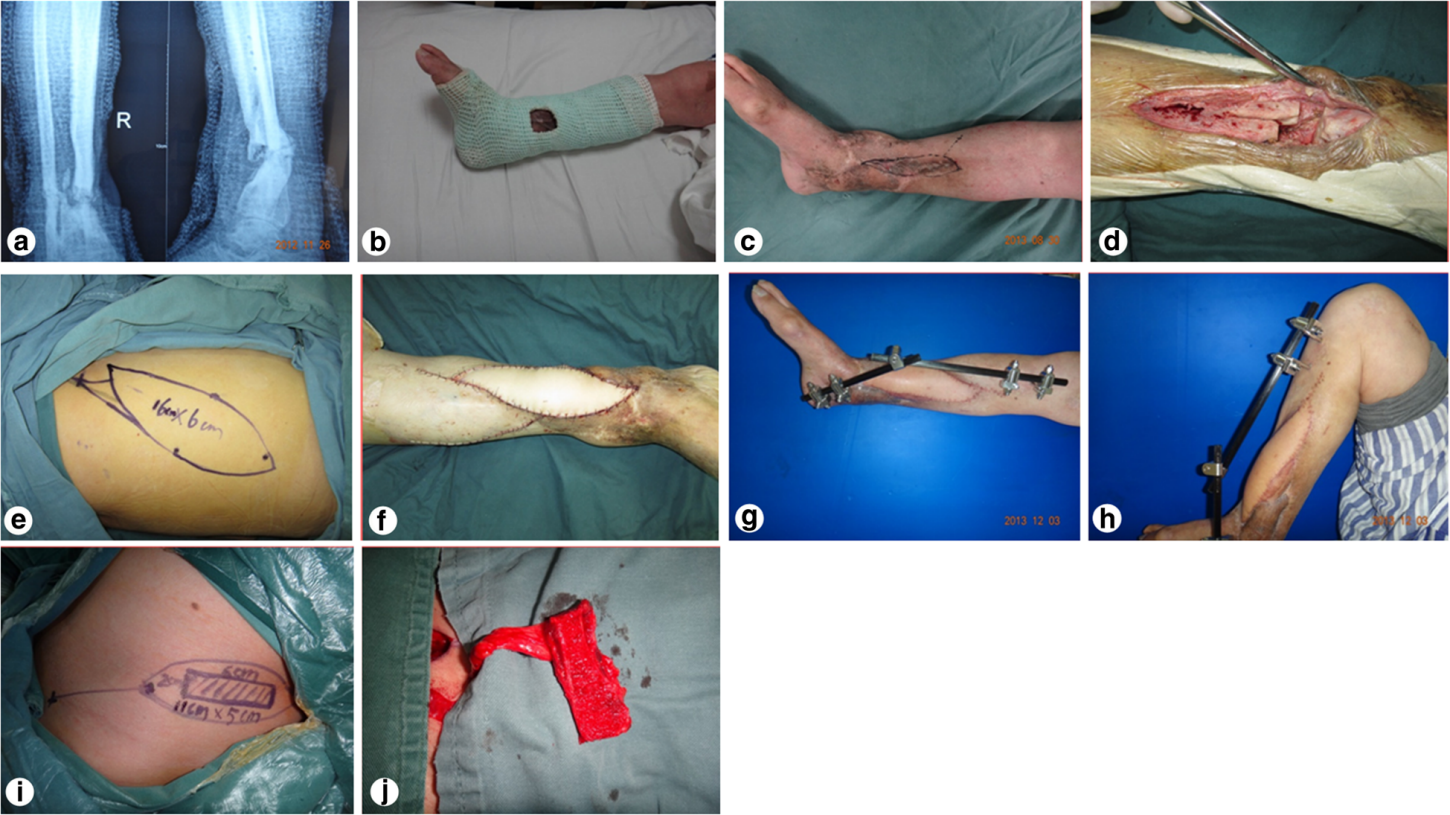
Bone fracture is not healed after removal of internal fixator (**a**). Fixation, fenestration, and dressing change (**b**). Sinus tract healing and bone scarring (**c**). Removal of the deactivated bone (**d**). Design of latissimus dorsi myocutaneous flaps (**e**). Postoperative repairing of latissimus dorsi myocutaneous flaps (**f**). Primary wound healing of latissimus dorsi myocutaneous flaps (**g**). Knee joint function (**h**). Design of iliac bone flaps (**i**). Cutting of iliac bone flaps (**j**)

**Fig. 6:**
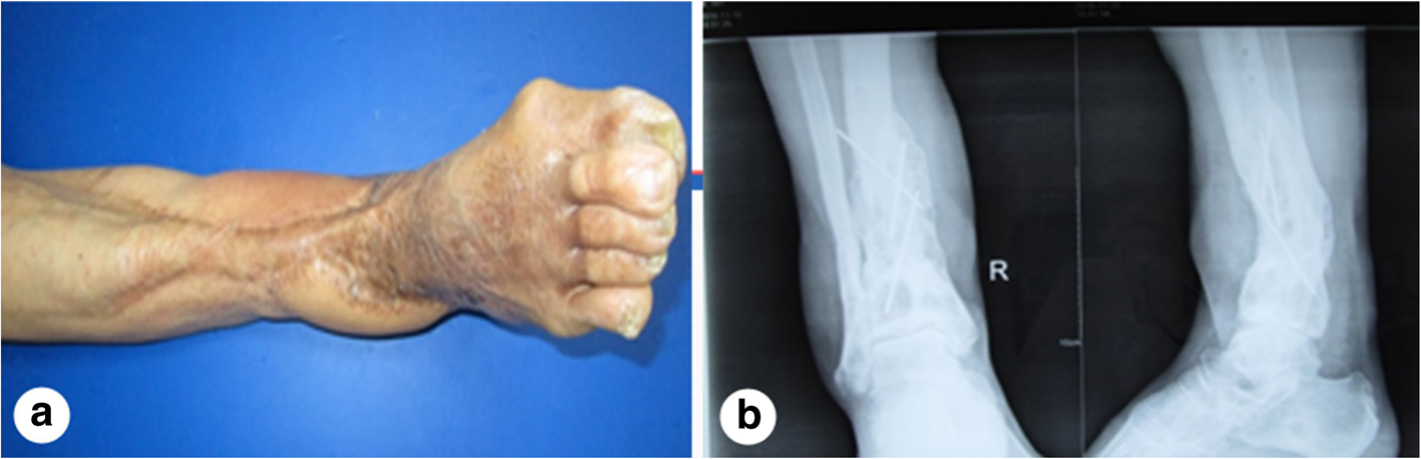
Wound surface healing after bone grafting (**a**). Bone graft healing (**b**)

**Fig. 7:**
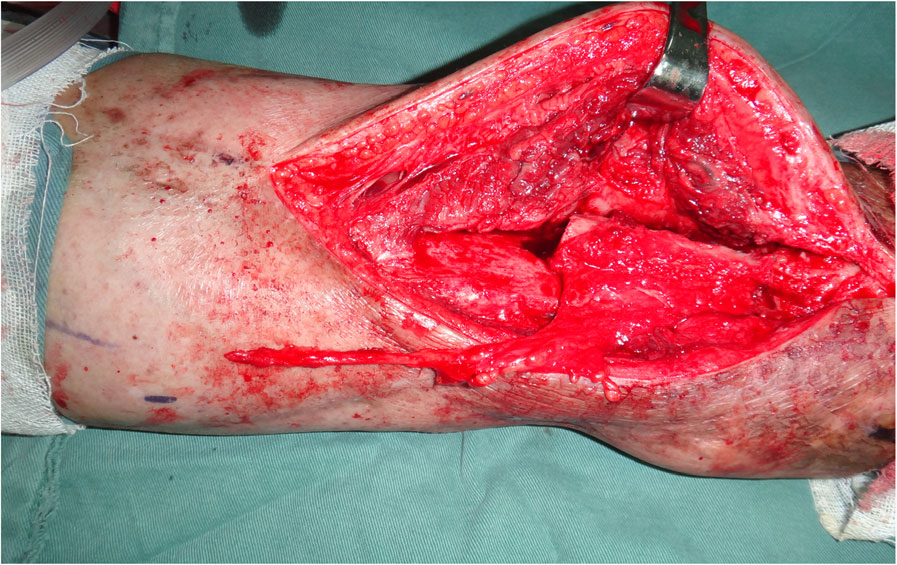
The skin window chamber of the inserted iliac crest

A male patient, aged 51 years, was admitted to our hospital due to a lower extremity injury. He was subsequently diagnosed with anterior and posterior tibial arterial injury, tibiofibular comminuted fracture, and avulsed wound of the skin soft tissues, as illustrated in Fig. 1. After admission, internal fixation, stent external fixation, vascular neuroanastomosis, and skin avulsion grafting were subsequently performed in the emergent department, as shown in Fig. 2. Postoperatively, partial skin necrosis was observed in the ankle and the steel plate was exposed (Fig. 3a). Then, lateral femoral flap grafting was delivered, as illustrated in Fig. 3b. Primary flap healing was obtained. The wound surface and puncturing site were dried at 6 months after the flaps were healed, and no significant exudates were observed, as shown in Fig. 3c. X-ray demonstrated that the bone fracture was partially healed and partial bone absorption was detected. The external fixation stent was removed, whereas the internal fixator was retained, as illustrated in Fig. 3d. At 1.5 years after injury, two sinus tracts with slight yellow exudates were seen in the middle and lower segments of the lower extremity (Fig. 4a). CT scans revealed that the bone mineral density was decreased and the bone fracture was not healed. After dressing change, the internal fixator was removed and dressing change was administered, as shown in Fig. 4b. Delayed wound healing was achieved after multiple symptomatic treatment and fixed by using plaster cast. After 6 months, the bone scarring was observed and the bone defects were not healed. The bone scars and necrotic tibiofibular bone tissues were debrided. The latissimus dorsi myocutaneous flaps were utilized to cover the wound surface and fill in the bone defects. The external fixation stent was retained. Primary wound healing was obtained in the wound surface and donor site, and no evident sinus tract was observed. The iliac bone flap grafting was delivered after 6 months. Primary wound healing was obtained in the donor site. X-ray during postoperative follow-up demonstrated that the bone defects were properly healed. The external fixation stent was removed following 6 months and fixed by using plaster cast (Fig. 5). At 8 months, the plaster cast fixation was removed and the patient was required to perform function training and exercise using the walking stick, as illustrated in Fig. 6. The situs with the inserted iliac crest flap was illustrated in Fig. 7.

## Discussion

Chronic osteomyelitis is evolved and aggravated from inappropriate or delayed treatment of acute osteomyelitis [12]. During the therapeutic procedures, sinus tract, skin scars and defects, and bone fracture and defects can spontaneously occur [13, 14]. Consequently, the granulation tissues, necrotic bone, and dead cavity should be thoroughly debrided to enhance the regional and systemic blood supply. In this investigation, 18 patients diagnosed with chronic osteomyelitis of the lower extremity undergoing stage I latissimus dorsi myocutaneous flap combined with stage II iliac bone flap grafting were recruited. High clinical efficacy and safety of the combined surgical interventions were obtained.

The latissimus dorsi belongs to the type V muscle. The blood supply of latissimus dorsi is dominantly controlled by the dorsal chest vessels and secondary segmental intercostal blood vessels. Zhao et al. [15] have demonstrated that latissimus dorsi myocutaneous flap can be widely applied to treat traumatic bone trauma, exposure of internal fixator, extensive tissue defects, deep wound filling and dynamic reconstruction after joint injury, etc. Usage of muscular flaps enhances the anti-infection capability of the affected patients. Tang et al. [16] have made certain modifications regarding the conventional latissimus dorsi myocutaneous flaps by enlarging the area of latissimus dorsi myocutaneous flaps and mitigating the severity of donor site injury. Previous study [17] has suggested that latissimus dorsi myocutaneous flap grafting accompanied with T-shaped arterial pedicle can restore the continuity of the blood supply arteries in the donor site and decrease the risk of disorders of the affected limbs. Usage of T-shaped blood vessels can perform vascular graft bridging, resolve the vascular defects in the donor site, increase the distal blood supply of the affected limbs, enhance regional blood supply, and lower the risk of bacterial infection, thereby effectively treating the chronic osteomyelitis.

The iliac bone has been widely recognized as the optimal donor site for autologous bone grafting [18]. The iliac ala is relatively smooth, wide, and thick, which yields mild postoperative complications. The blood supply of the iliac ala originates from the deep and shallow iliac arteries and the ascending branches of the lateral femoral circumflex artery.

In this clinical trial, latissimus dorsi myocutaneous flap anastomosis was initially performed, and subsequently, the arterial and venous pedicles of the latissimus dorsi myocutaneous flaps were cut during the secondary surgery and anastomosed to the vascular pedicles of the iliac bone flaps. Two patients with osteomyelitis of the lower segment of the femur and 4 cases with osteomyelitis of the tibial plateau utilized descending genicular artery-saphenous artery as the blood supply arteries. The descending genicular artery penetrated through the adductor canal along with the superficial femoral artery and extended downwards to the saphenous artery [19, 20]. In this investigation, 6 patients underwent saphenous artery exploration, and descending genicular arteries were the migrating vessels rather than the descending genicular artery. In total, 12 patients were diagnosed with chronic osteomyelitis of the lower segment of the tibia. Among them, 5 cases had anterior tibial arterial and accompanying venous anastomosis and 7 received posterior tibial arterial and accompanying venous anastomosis. Nine patients underwent bridging anastomosis and 3 were subjected to direct anastomosis to the anterior tibial artery and accompanying vein.

All the latissimus dorsi myocutaneous flaps survived in 18 patients. After the corresponding surgery, primary wound healing was achieved in 11 patients, delayed wound healing was obtained in 7 cases. All wounds were completely healed with postoperative 2 months. Following the iliac bone flap grafting, primary wound healing was accomplished in all cases. All dorsal window chambers survived. The bone defects were properly restored within 4–12 postoperative months. Paley’s criterion was employed to evaluate the bone healing and function recovery of the lower limbs. Excellent bone healing was accomplished in 13 patients and good bone healing was obtained in the remaining 5 cases. In terms of the function recovery, 12 patients obtained excellent effect, 5 had good effect, and 1 obtained moderate effect.

In this study, all 18 patients underwent multiple cycles of debridement, lavage, vacuum sealing drainage (VSD), intraoperative removal of necrotic bone, and bacterial culture as well as administration of sensitive antibiotics for anti-infection treatment. However, 3 cases with chronic osteomyelitis of the lower segment of the tibia presented with preoperative multidrug resistant bacterial infection. During the latissimus dorsi myocutaneous flap grafting, the marrow cavity was filled with muscular sleeve and broad-spectrum antibiotics were administered after surgery. Eventually, primary wound healing was obtained in these 3 patients. Usage of sensitive antibiotics does not play a decisive role, whereas application of anti-infection interventions and improvement of regional blood vessels are the key procedures. This conclusion remains to be further elucidated and validated by subsequent investigations with large sample size. In addition, previous researchers have reported that the recurrence of the osteomyelitis after bisphosphonate-related osteonecrosis of the jaw is a manifestation in a microvascular iliac bone flap [21]. Consequently, the long-term recurrence issues remain to be further elucidated by long-term clinical trials.

Kirschner wire can be adopted for internal fixation and plaster for external fixation. Although chronic osteomyelitis was covered by muscular flaps and the bacterial infection was controlled, the risk of chronic osteomyelitis recurrence was still high. Consequently, Kirschner wire combined with plaster external fixation is recommended to reduce the risk of infection and mitigate the economic burden of the patients as possible. Besides, functional training is also of great necessity. Postoperative, the patients should be encouraged to receive and complete systematic functional exercises in the Rehabilitation Department, which can enhance the clinical efficacy and lower the risk of postoperative complications.

Prior to the surgery, the surgeons and nurses are obliged to deliver psychological interventions to ease the stress of the patients, inform them with the specific procedures of the surgery, and encourage them to get fully prepared and actively cooperate with the surgical procedures. In this investigation, 18 enrolled patients were explicitly informed with the surgical details, therapeutic regime, potential risk, and therapeutic cycle before the surgery. All patients were fully prepared and actively cooperated with the surgeons to successfully complete the treatment. High clinical efficacy and safety were obtained during postoperative follow-up.

## Conclusion

This combined surgical technique has several advantages. First, the latissimus dorsi myocutaneous flaps possess abundant blood supply, large vascular diameter, wide cutting range, and relatively concealed donor site. Second, latissimus dorsi myocutaneous flaps are accompanied with muscular tissues and are highly resistant to bacterial infection. It can remove the dead cavity and improve regional blood supply. Third, iliac bone flaps possess reliable blood supply, which can accelerate bone healing. Fourth, iliac bone flaps carry skin window chamber, which contributes to blood supply, enhance the cosmetic appearance of the wound surface, and remove the bone scars. Fifth, vascular pedicles of latissimus dorsi myocutaneous flaps were utilized as the vessel pedicles of the iliac bone flaps for anastomosis at a low position, which contributes to intraoperative vascular anastomosis. Sixth, the affected limbs can be restored after different stages of clinical interventions. There are several limitations to be acknowledged. First, the cycle of treatment is relatively long. Second, repeated surgeries increase the medical expense. Third, surgical scars are induced at multiple sites, which yield poor cosmetic appearance. Finally, the time of plaster fixation is relatively long, which affects the joint motion and exercise.
